# Expanded Classification of Hepatitis C Virus Into 7 Genotypes and 67 Subtypes: Updated Criteria and Genotype Assignment Web Resource

**DOI:** 10.1002/hep.26744

**Published:** 2013-12-20

**Authors:** Donald B Smith, Jens Bukh, Carla Kuiken, A Scott Muerhoff, Charles M Rice, Jack T Stapleton, Peter Simmonds

**Affiliations:** 1 Centre for Immunity, Infection and Evolution, University of EdinburghScotland, UK; 2 Copenhagen Hepatitis C Program (CO-HEP), Department of Infectious Diseases and Clinical Research Centre, Copenhagen University Hospital, Hvidovre, and Department of International Health, Immunology and Microbiology, Faculty of Health and Medical Sciences, University of CopenhagenDenmark; 3 Theoretical Biology and Biophysics group, Los Alamos National LaboratoryLos Alamos, NM, USA; 4 Abbott Diagnostics Research and DevelopmentAbbott Park, IL, USA; 5 Laboratory of Virology and Infectious Disease, Center for the Study of Hepatitis C, The Rockefeller UniversityNew York, NY, USA; 6 Medical Service, Iowa City Veterans Affairs Medical Center, Departments of Internal Medicine and Microbiology, University of IowaIowa City, IA, USA

## Abstract

The 2005 consensus proposal for the classification of hepatitis C virus (HCV) presented an agreed and uniform nomenclature for HCV variants and the criteria for their assignment into genotypes and subtypes. Since its publication, the available dataset of HCV sequences has vastly expanded through advancement in nucleotide sequencing technologies and an increasing focus on the role of HCV genetic variation in disease and treatment outcomes. The current study represents a major update to the previous consensus HCV classification, incorporating additional sequence information derived from over 1,300 (near-)complete genome sequences of HCV available on public databases in May 2013. Analysis resolved several nomenclature conflicts between genotype designations and using consensus criteria created a classification of HCV into seven confirmed genotypes and 67 subtypes. There are 21 additional complete coding region sequences of unassigned subtype. The study additionally describes the development of a Web resource hosted by the International Committee for Taxonomy of Viruses (ICTV) that maintains and regularly updates tables of reference isolates, accession numbers, and annotated alignments (http://talk.ictvonline.org/links/hcv/hcv-classification.htm). The Flaviviridae Study Group urges those who need to check or propose new genotypes or subtypes of HCV to contact the Study Group in advance of publication to avoid nomenclature conflicts appearing in the literature. While the criteria for assigning genotypes and subtypes remain unchanged from previous consensus proposals, changes are proposed in the assignment of provisional subtypes, subtype numbering beyond “w,” and the nomenclature of intergenotypic recombinant. *Conclusion*: This study represents an important reference point for the consensus classification of HCV variants that will be of value to researchers working in clinical and basic science fields. (Hepatology 2014;59:318-327)

Soon after the publication of the first nearly complete genome sequence of hepatitis C virus (HCV) in 1989,^1^ it became apparent that isolates from different individuals or countries showed substantial genetic diversity. After much research and surveying by groups worldwide, this variation was summarized and variants assigned as genotypes and subtypes in a consensus classification and nomenclature system and formal rules were agreed for the assignment and naming of future variants.^2^ Genotype and subtype assignments required: (1) one or more complete coding region sequence(s); (2) at least three epidemiologically unrelated isolates; (3) a phylogenetic group distinct from previously described sequences; (4) exclusion of intergenotypic or intersubtypic recombination, whether the components were classified or not.

The application of these criteria confirmed the assignment of six distinct genotypes, comprising 18 subtypes. In addition, 58 subtypes were provisionally assigned pending the availability of a complete coding region sequence or additional isolates. This agreement on nomenclature was mirrored by the establishment of several curated databases that organized HCV sequences as they became available and indicated which genotypes and subtypes were confirmed or provisionally assigned (Los Alamos HCV Sequence Database,^3^ euHCVdb,^4^ Hepatitis Virus Database: http://s2as02.genes.nig.ac.jp/). Concurrently, a proposal was made to unify the numbering of HCV with reference to the genotype 1a isolate H77 (AF009606).^5^

Recently, this remarkable agreement and cooperation in HC>V nomenclature has been complicated by several developments. None of the HCV sequence databases are now actively curated and responsibility for naming new genotypes and subtypes has reverted *de facto* to individual researchers. This, combined with publication delays, has created new contradictions in which isolates assigned to the same subtype (4b: FJ462435, FJ025855, FJ025856, and FJ025854; 6k: DQ278891 and DQ278893; 6u: EU408330, EU408331, and EU408332) belong to different subtypes according to the consensus criteria.^2^ Another challenge is that the number of complete coding region sequences has increased from 238 in 2005 to more than 1,300. Similarly, the number of variants matching the criteria for assignment as confirmed genotypes/subtypes has expanded from 18 to 67; several recent publications contain figures that are illegible with regard to isolate name and/or accession number,^6^–^10^ complicating subsequent comparisons.

Finally, advances in sequencing technology have accelerated the rate at which HCV sequences are generated. Recent articles have reported the partial sequences of 282 isolates from Vietnam^11^ and 393 isolates from China,^10^ in each case identifying additional subtypes of genotype 6. Technological advances have also made it easier to obtain HCV complete coding region sequences through both dideoxysequencing and pyrosequencing. The latter technique was recently used to obtain 31 complete coding region sequences belonging to 13 different subtypes.^8^ More than 225,000 HCV sequences are now available on GenBank and about 30,000 added every year. This volume of sequence information and the diversity of known HCV variants make it increasingly important for researchers to have a single curated resource to refer to for accurate subtype designations, reference genomes and alignments.

This article updates the genotype and subtype assignments^2^,^7^ and the nomenclature rules, and describes the establishment of a reference Website hosted by the International Committee for the Taxonomy of Viruses (ICTV) to validate new genotype and subtype assignments, and provide updated reference alignments.

## Revision of Confirmed Genotypes and Subtypes

**Fig. 1:**
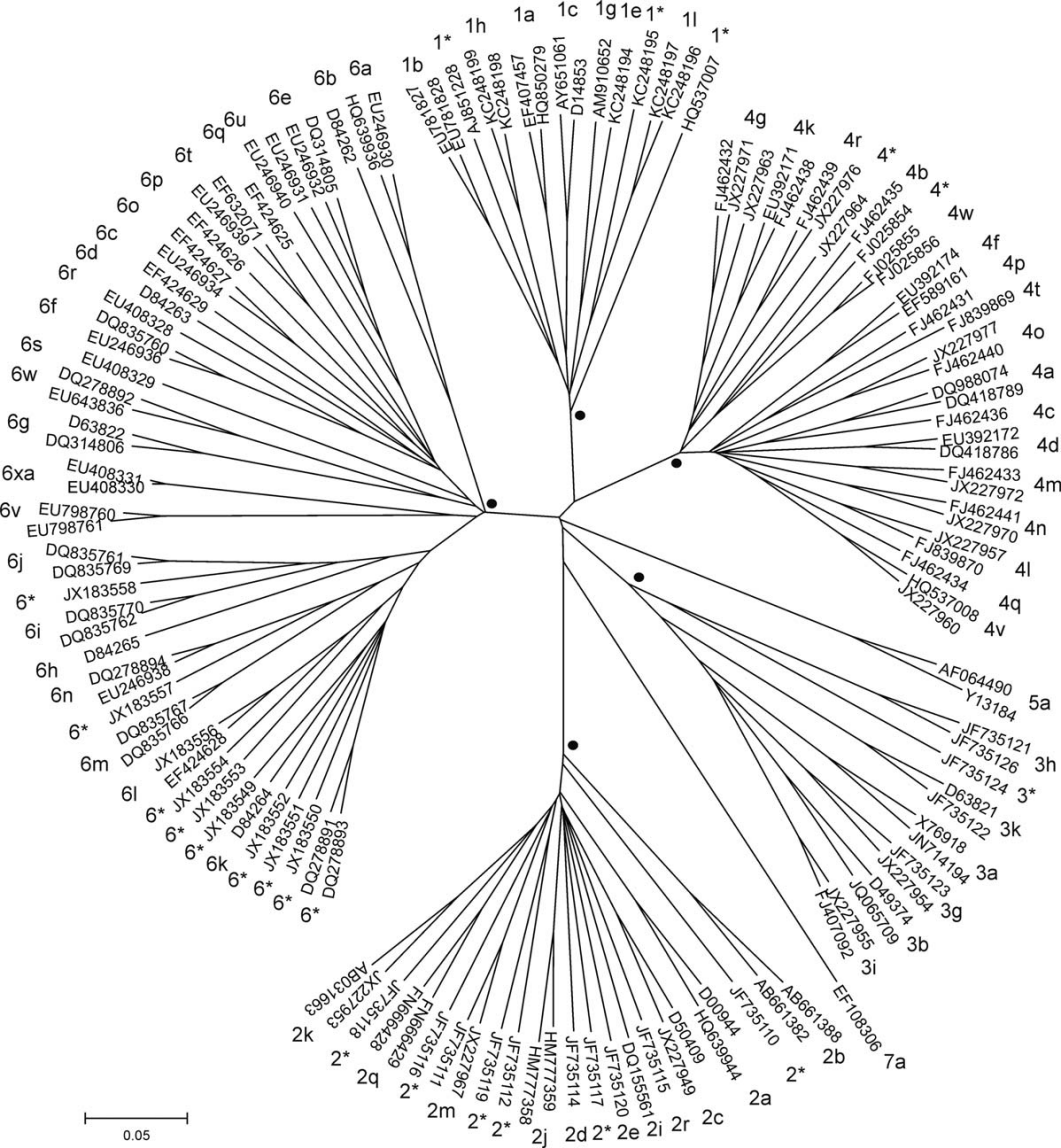
Phylogenetic tree of 129 representative complete coding region sequences. Up to two representatives of each confirmed genotype/subtype were aligned (together with a third extreme variant of subtypes 4g and 6e) and a neighbor joining tree constructed using maximum composite likelihood nucleotide distances between coding regions using MEGA5.^83^ Sequences were chosen to illustrate the maximum diversity within a subtype. Tips are labeled by accession number and subtype (*unassigned subtype). For genotypes 1, 2, 3, 4, and 6, the lowest common branch shared by all subtypes and supported by 100% of bootstrap replicates (n = 1,000) is indicated by ·.

Unique HCV complete or nearly complete coding region sequences available on NCBI Genome (969 sequences, http://www.ncbi.nlm.nih.gov/genome) and the Los Alamos HCV sequence database (1,364 sequences >8,000 nt from http://hcv.lanl.gov/content/index) were aligned within SSEv1.1^12^ using Muscle v3.8.31^13^ and refined manually. Phylogenetic analysis of sequences containing >95% of the coding region reveals seven major phylogenetic groupings corresponding to genotypes 1-7 (Fig. 1). Within these genotypes, grouping of the constituent subtypes is supported by 100% of bootstrap replications.

**Fig. 2:**
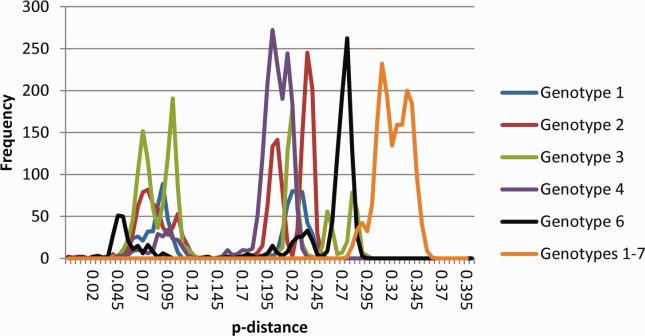
Distribution of p-distances between complete coding region sequences. The frequency of p-distances was calculated within and between genotypes using SSE.^12^ Intra-genotype pairwise distances were calculated for all available complete coding region sequences except for subtypes 1a, 1b, and 2b where 20 random sequences were used. For p-distances >0.15 (equivalent to a percent difference of 15%), frequencies were scaled to reduce the maximum frequency to less than 300. Distances between genotypes were calculated using one or two representatives of each confirmed and unassigned subtype, with the frequencies scaled as above.

Based on the consensus criteria,^2^ confirmed subtypes (indicated by a letter following the genotype) require a complete or nearly complete coding region sequence differing from other sequences by at least 15% of nucleotide positions and sequence information from at least two other isolates in core/E1 (>90% of the sequence corresponding to positions 869 to 1,292 of the H77 reference sequence [accession number AF009606] numbered according to reference^5^) and NS5B (>90% of positions 8,276 to 8,615) (Table 1). The use of a 15% threshold over the complete coding region is supported by analysis of the large number of potential subtypes now sequenced (Fig. 2). This reveals major and consistently placed gaps in the distribution of pairwise distances between and within subtypes of each genotype as follows: genotype 1: 12.9%-17.0%, genotype 2: 13.1%-17.6%, genotype 3: 12.5%-19.6%, genotype 4: 12.7%-15.3% (except distances of 14% and 14.2% between JX227963 and two subtype 4g sequences), and genotype 6: 9.9%-14.9% (except distances of 13.1%-13.7% between EU246931 and three subtype 6e sequences). Hence, for all genotypes and with remarkably few exceptions, a clear division can be made between isolates that differ by <13% over their complete coding region sequences (members of the same subtype) and those that differ by >15% (different genotypes or subtypes). This analysis includes sequences distinct from any of the confirmed HCV subtypes but not currently represented by three or more independent isolates that remain unclassified subtypes (Table 2). Whether the exceptions noted are due to technical problems or to differing epidemiological histories is unknown.

**Table 1:** Confirmed HCV Genotypes/Subtypes

Genotype*	Locus/Isolate(s)†	Accession number(s)	Reference(s)
*Genotype 1*			
1a	HPCPLYPRE, HPCCGAA	M62321, M67463	29,30
1b	HPCJCG, HPCHUMR	D90208, M58335	31,32
1c	HPCCGS, AY051292	D14853, AY051292	33
**1e**	**148636**	**KC248194**	9
**1g**	**1804**	**AM910652**	34
**1h**	**EBW443, EBW9**	**KC248198, KC248199**	9
**1l**	**136142, EBW424**	**KC248193, KC248197**	9
*Genotype 2*			
2a	HPCPOLP, JFH-1	D00944, AB047639	35,36
2b	HPCJ8G, JPUT971017	D10988, AB030907	37,38
2c	BEBE1	D50409	39
**2d**	**QC259**	**JF735114**	40
**2e**	**QC64**	**JF735120**	40
**2i**	**D54**	**DQ155561**	41
**2j**	**C1799, QC232**	**HM777358 JF735113**	6,40
2k	VAT96	AB031663	42
**2m**	**QC178, BID-G1314**	**JF735111, JX227967**	40,8
**2q**	**963, 852**	**FN666428, FN666429**	43
**2r**	**QC283**	**JF735115**	40
*Genotype 3*			
3a	HPCEGS, HPCK3A	D17763, D28917	44,45
3b	HPCFG	D49374	46
**3g**	**BID-G1243, QC260**	**JX227954, JF735123**	8,21
**3h**	**QC29**	**JF735121**	21
**3i**	**IND-HCV, BID-G1244**	**FJ407092, JX227955**	8
3k	HPCJK049E1, **QC105**	D63821, **JF735122**	47,21
*Genotype 4*			
4a	ED43	Y11604	48
**4b**	**QC264**	**FJ462435**	16
**4c**	**QC381**	**FJ462436**	16
**4d**	**03-18, QC382**	**DQ418786, FJ462437**	49,16
**4f**	**IFBT88, PS6**	**EF589161, EU392175**	50,51
**4g**	**QC193**	**FJ462432**	16
**4k**	**PS3, QC383**	**EU392173, FJ462438**	51,16
**4l**	**QC274**	**FJ839870**	16
**4m**	**QC249**	**FJ462433**	16
**4n**	**QC97**	**FJ462441**	16
**4o**	**QC93**	**FJ462440**	16
**4p**	**QC139**	**FJ462431**	16
**4q**	**QC262**	**FJ462434**	16
**4r**	**QC384**	**FJ462439**	16
**4t**	**QC155**	**FJ839869**	16
**4v**	**CYHCV073, BID-G1248**	**HQ537009, JX227959**	52,8
**4w**‡	**P212, P245**	**FJ025855, FJ025856**	14
*Genotype 5*			
5a	EUH1480, SA13§	Y13184, AF064490	53,54
*Genotype 6*			
6a	EUHK2,6a33	Y12083, **AY859526**	55,56
6b	Th580	D84262	57
**6c**	**Th846**	**EF424629**	58
6d	VN235	D84263	57
**6e**	**GX004**	**DQ314805**	59
**6f**	**C-0044**	**DQ835760**	60
6g	HPCJK046E2	D63822	47
6h	VN004	D84265	57
**6i**	**Th602**	**DQ835770**	60
**6j**	**Th553**	**DQ835769**	60
6k	VN405	D84264	57
**6l**	**537796**	**EF424628**	58
**6m**	**B4/92**	**DQ835767**	60
**6n**	**KM42, D86/93**	**DQ278894, DQ835768**	17,60
**6o**	**QC227**	**EF424627**	58
**6p**	**QC216**	**EF424626**	58
**6q**	**QC99**	**EF424625**	58
**6r**	**QC245**	**EU408328**	61
**6s**	**QC66**	**EU408329**	61
**6t**	**VT21, D49**	**EF632071, EU246939**	62,19
**6u**	**D83**	**EU246940**	19
**6v**	**NK46, KMN-02**	**EU158186, EU798760**	62,63
**6w**	**GZ52557, D140**	**DQ278892, EU643834**	17,64
**6xa**‖	**DH012, DH028**	**EU408330, EU408332**	18
***Genotype 7***			
**7a**	**QC69**	**EF108306**	

Additions and changes from assignments proposed in 2 shown in **bold**.

* Consensus proposed genotype/subtype names. Where multiple sequences of a HCV genotype are available, two sequences have been listed, prioritized by (a) publication date or (b) submission date when unpublished.

† Locus (or isolate name if locus is the same as the accession number).

‡ Previously described as 4b.^7^,^14^

§ Sequence obtained from acute phase plasma of a chimpanzee experimentally infected with (human-derived) isolate SA13.

‖ Previously described as 6u.^18^

**Table 2:** Unassigned Complete Coding Region Sequences

Genotype*	Locus/Isolate(s)†	Accession no(s)	Reference
*Genotype 1*			
1_AJ851228	AJ851228	AJ851228	65
1_KC248195	160526	KC248195	9
1_ HQ537007	CYHCV025	HQ537007	52
*Genotype 2*			
2_JF735119	QC331	JF735119	40
2_JF735112	QC182	JF735112	40
2_JF735110	QC114	JF735110	40
2_JF735117	QC297	JF735117	40
2_JF735116	QC289	JF735116	40
2_JF735118	QC302	JF735118	40
*Genotype 3*			
3_JF735124	QC115	JF735124	21
*Genotype 4*			
4_JX227964	BID-G1253	JX227964	8
4_FJ025854‡	P026	FJ025854	14
*Genotype 6*			
6_DQ278891§	KM45,KM41	DQ278891,DQ278893	17
6_JX183550	QC273	JX183550	20
6_JX183552	TV476	JX183552	20
6_JX183549	KM35	JX183549	20
6_JX183551	TV257	JX183551	20
6_JX183553	TV533	JX183553	20
6_JX183554	L349	JX183554	20
6_JX183557	DH027	JX183557	20
6_JX183558	QC271	JX183558	20

* Classification of sequences into genotypes but without subtype assignments using the format “genotype_Accession number.”

† Locus (or isolate name if locus is the same as the accession number).

‡ Previously described as 4b.^14^

§ Previously described as 6k.^17^

The seven confirmed genotypes (discussed below) comprise 67 confirmed subtypes, 20 provisionally assigned subtypes, and 21 unassigned subtypes. These tables have been posted on the ICTV Website at http://talk.ictvonline.org/links/hcv/hcv-classification.htm and will be updated regularly by the authors with information shared across existing HCV databases (http://hcv.lanl.gov/; http://euhcvdb.ibcp.fr/euHCVdb/), typing tools, and other resources (e.g., http://www.bioafrica.net/rega-genotype/html/subtypinghcv.html; http://comet.retrovirology.lu/; http://hcv.lanl.gov/content/sequence/phyloplace/; http://s2as02.genes.nig.ac.jp/; http://www.viprbrc.org/). Alignments including representatives of these subtypes are available on the ICTV Website and at http://hcv.lanl.gov/content/sequence/NEWALIGN/align.html/.

The process of producing these tables has detected a small number of variants with conflicting assignments. Isolates P026, P212, P245, (FJ025854-6) are described as subtype 4b,^14^ but these complete coding region sequences show <85% identity to the core/E1 of isolate Z1 (U10235, L16677), provisionally assigned as 4b^15^ that is more closely related to core/E1 of the complete coding region sequence of isolate QC264 (FJ462435^16^). P212 and P245 belong to the same, novel subtype for which NS5B sequence is available from a third isolate (P213, GU049362), so this becomes confirmed subtype 4w. Isolate P026 differs from all other genotype 4 sequences by >17.5% but being represented by a single sequence remains currently unassigned (Table 2).

Similarly, isolates KM45 and KM41 (DQ278891,3) have been assigned to subtype 6k,^17^ but differ by >17% in complete coding region sequence from the subtype 6k isolate VN405 (D84264) and 6.7% from each other, and so remain an unclassified subtype of genotype 6. Two distinct groups of isolates have been assigned to subtype 6u; EU408330-2^18^ and EU246940.^19^ The latter was submitted first to GenBank and is represented by NS5B sequences from two additional isolates and so is assigned subtype 6u, while EU408330, EU408331, and EU408332 are designated subtype 6xa (see below).

Finally, our analysis of both phylogenetic groupings and sequence distances suggests that a number of isolates^20^ described in their GenBank accessions as “subtype k-related” (QC273, TV257, TV476, KM35), “subtype l-related” (TV533, L349), “intermediate between subtypes 6m and 6n” (DH027), or “intermediate between subtypes 6j and 6i” (QC271) should be considered as unassigned novel subtypes.

## Additional Taxonomic Levels

In making this taxonomic distinction into virus genotypes and subtypes we are aware of the difficulties of imposing a discrete classification scheme on a complex taxonomy. In particular, for genotypes 3 and 6 there are undoubtedly several hierarchies of taxonomic relationships. For example, subtypes 6k and 6l form a clade along with several unassigned genotype 6 isolates.^20^ A higher-level clade includes these sequences and subtypes 6m and 6n, while a further grouping consists of these subtypes and subtypes 6i and 6j (Fig. 1). These phylogenetic hierarchies are reflected in the discontinuous distribution of p-distances between complete coding region sequences (Fig. 2), which comprises three almost merging distributions (roughly 15% to 20%, 20% to 25%, and 25% to 30%). Three distributions of intersubtype distances were also observed for genotype 3 (20% to 25%, 25% to 27%, and 27% to 30%), two distributions for genotype 2 (18% to 22.5%, 23% to 26.5%), and uniform distributions for genotype 1 (17.7% to 25.4%) and genotype 4 (15.3% to 23.1%). However, the internal divisions defined by the multiple distributions of distances within genotypes 2, 3, and 6 have not been shown to correspond with geographical or epidemiological differences. The higher-level grouping of subtypes 3b, 3g, and 3i does not reflect a common geographical origin distinct from that of 3h and 3k.^21^ There is also no geographical correlation with the groupings of subtypes 6k, 6l, and various unassigned isolates; for 6m, 6n, and an unassigned isolate; for 6h, 6i, 6j, and an unassigned isolate; for 6a and 6b; for 6f and 6r; or for 6r and 6e.^22^ Similarly, there are currently no known virological or clinical reasons to recognize these higher-level groupings. Without practical utility, we therefore propose that the observed within-genotype hierarchies are not given any formal recognition in their nomenclature.

## Proposed Updates and Changes to Rules for Genotype/Subtype Assignments

### Subtype Names

By definition, subtype name assignments would be limited to a maximum of 26 if designated by a single letter suffix (e.g., 2a-2z). We therefore suggest that subtypes are assigned up to the letter “w” and subsequent designations follow the eXtended form xa, xb, … xz, in turn followed by ya, … yz, za, … zz, potentially giving a total of 101 subtypes of each genotype. This avoids potentially ambiguous terms such as “subtype 6x,” which could be interpreted as “genotype 6 of unknown subtype,” or designations such as “subtype 3aa,” which might suggest a relationship with 3a.

### Provisional Genotypes

According to the 2005 consensus classification protocol^2^ new genotypes could be provisionally assigned from a single complete coding region sequence, but partial or complete coding region sequences from additional isolates would be required to confirm these assignments. Since then only one provisional genotype has been identified (7a) represented by a single isolate (QC69, EF108306). Thus, in contrast to subtype assignments, the number of genotypes appears relatively limited and the requirement to sequence multiple isolates now seems over-onerous. We propose that only a single complete coding region sequence is needed to confirm a new genotype assignment; QC69 is therefore confirmed as genotype 7a.

### Provisional Subtypes

The 2005 consensus protocol also proposed that provisional subtypes could be assigned on the basis of sequence comparisons in the core/E1 and NS5B regions for at least three independent isolates, requiring in addition a complete coding region sequence before being confirmed. Of the 58 subtypes provisionally assigned in the 2005 article, 38 have now been confirmed (Table 1). However, it is now much easier to obtain complete coding region sequences and very few additional provisional subtypes have been proposed. Instead, some authors have inconsistently labeled unusual isolates with the suffix “?,” “unassigned group I”^11^,^23^ or “subtype 1(I).”^9^ We propose that provisional subtype designations should no longer be provided for variants where complete genome sequences are lacking. The 20 remaining provisionally assigned subtypes will be maintained (Table 3), since they already exist in the literature. Future subtype assignments will only be made (as confirmed assignments) when sequence data from three or more isolates including at least one complete or nearly complete coding region is provided. Where a complete coding region sequence is available but there are fewer than three isolates, we propose that these remain unassigned. In Table 2 these are labeled using the form “Genotype_Accession number,” e.g., 1_AJ851228.

**Table 3:** Remaining Provisionally Assigned HCV Subtypes

		Accession number(s)*		
			
	Isolate†^†^	Core/E1	NS5B	Reference(s)
*Genotype 1*				
1d	HC1-N15, HC1-N16	L39299, L39302	L38377, L38372	66
1f	FR2	L38350	L38371	66
1i	FR16, QC77	n.a., AY434119	L48495, AY434120	67,68
1j	QC2, QC89	AY434158, AY434128	AY434106, AY434129	67
1k	QC68, QC82	AY434112, AY434122	AY434113, AY434123	67
*Genotype 2*				
2f	JK081, JK139	D49754, D49757	D49769, D49777	47
2g	MED017	n.a.	X93323	69
2h	MED007	n.a.	X93327	69
2l	FR15	n.a.	L48494	68
2n	NL50	L39309	L44602	66
2o	FR4	L38333	L38373	66
2p	NL33	L39300	L44601	66
*Genotype 3*				
3c	NE048	D16612	D14198/D16613	70
3d	NE274	D16620	D14200/D16621	70
3e	NE145	D16618	D16619	70
3f	NE125, PK64	D16614, n.a.	D14203/D16615, L78842	70,71
*Genotype 4*				
4e	CAM600, GB809	L29589, L29629	L29590, L29626	72
4h	GB438, FrSSD35	L29610, n.a.	L29611, AJ291249	72,73
4i	CAR4/1205	L36439	L36437	74
4j	CAR1/501	n.a.	L36438	74

* Accession numbers of sequences from the core/E1 and NS5B regions. “n.a.”: not available; “/”: denotes that the core/E1 or NS5B sequences are available from two different accession numbers.

† Examples of each provisionally assigned HCV.

### Recombinant and Other Forms

One issue that was not addressed in the 2005 consensus protocol^2^ was the naming of the newly discovered recombinant forms of HCV, their importance being unknown. Nine different recombinant forms of HCV have now been described (Table 4), of which only one (2k/1b) is represented by multiple isolates; no multiple recombinants have been reported (reviewed in reference^24^). In this context it does not seem necessary to revise the nomenclature generally used in the literature in which “RF” (recombinant form) is followed by the contributory subtypes separated by “/” in the order in which they appear in the complete genome sequence. We suggest that recombinant forms with the same genotypic structure but with different breakpoints or where the component genomic sections are unrelated are numbered consecutively with a numerical suffix (for example, RF2b/1b_1).

**Table 4:** Recombinant (RF) HCV Complete Coding Region Sequences

RF*	Breakpoint†	Accession	Isolates‡	Reference
RF2k/1b	3186	AY587845	33	75–77
RF2i/6p	3405-3464	DQ155560	1	41
RF2b/1b_1	3456	DQ364460	1	78
RF2/5	3366-3389	AM408911	1	79
RF2b/6w	3429	EU643835	1	64
RF2b/1b_2	3432	AB622121	1	80
RF2b/1a	3429-3440	JF779679	1	81
RF2b/1b_3	3286-3293	AB677530	1	82
RF2b/1b_4	3286-3293	AB677527	1	82

* Recombinant forms (RF) for which complete genome sequences are available are named according to the subtypes from which they are derived and in the order in which these appear in the genome.

† Breakpoints are numbered with reference to H77 (AF009606).

‡ Number of individuals from whom the RF has been isolated.

### Proposals for New Genotype/Subtype Assignments

The ICTV Flaviviridae Study Group is willing to take a coordinating role in the assignment of newly described variants of HCV. We urge researchers who have characterized new HCV variants that potentially qualify as new types or subtypes to contact Donald Smith (D.B.Smith@ed.ac.uk) or any member of the Study group (listed on http://ictvonline.org/subcommittee.asp?committee=25&se=5) in confidence before publication so that naming conflicts can be avoided and appropriate assignments made.

## Future Developments

Despite the increasing number and diversity of HCV sequences, the system of classification of variants into genotypes and subtypes has proven surprisingly robust. The seven confirmed genotypes have strong bootstrap support (Fig. 1), and the partition of these genotypes into subtypes that differ over a complete coding region sequence by >15% reflects a natural hiatus in the distribution of sequence distances (Fig. 2). We welcome any comments or suggestions for the proposed classification guidelines. Areas of uncertainty remain with respect to the region of endemicity of genotype 5, represented by a single subtype isolated in Europe, Brazil, North Africa, and South Africa, and genotype 7, isolated from an emigrant from the Congo. We might also anticipate the further discovery of other HCV-like viruses in the genus *Hepacivirus*,^25^–^28^ and variants closer genetically to HCV than the nonprimate hepacivirus that appears to be an endemic infection of horses worldwide.^25^ As more is learned about the host-specificity and diversity of hepaciviruses, the genotype classification of HCV may be logically incorporated within a unified classification of hepaciviruses at the species and potentially subspecies and subgenus levels.

## Abbreviations

HCV: hepatitis C virus
ICTV: International Committee for Taxonomy of Viruses

## References

[1] Choo Q, Kuo G, Weiner A, Overby L, Bradley D, Houghton M (1989). Isolation of a cDNA clone derived from a blood-borne non-A, non-B viral hepatitis genome. Science.

[2] Simmonds P, Bukh J, Combet C, Deléage G, Enomoto N, Feinstone S (2005). Consensus proposals for a unified system of nomenclature of hepatitis C virus genotypes. Hepatology.

[3] Yusim K, Richardson R, Tao N, Dalwani A, Agrawal A, Szinger J (2005). Los alamos hepatitis C immunology database. Appl Bioinformatics.

[4] Combet C, Garnier N, Charavay C, Grando D, Crisan D, Lopez J (2007). euHCVdb: the European hepatitis C virus database. Nucleic Acids Res.

[5] Kuiken C, Combet C, Bukh J, Shin-I T, Deleage G, Mizokami M (2006). A comprehensive system for consistent numbering of HCV sequences, proteins and epitopes. Hepatology.

[6] Sulbarán MZ, Di Lello FA, Sulbarán Y, Cosson C, Loureiro CL, Rangel HR (2010). Genetic history of hepatitis C virus in Venezuela: high diversity and long time of evolution of HCV genotype 2. PLoS One.

[7] Nakano T, Lau GMG, Lau GML, Sugiyama M, Mizokami M (2012). An updated analysis of hepatitis C virus genotypes and subtypes based on the complete coding region. Liver Int.

[8] Newman RM, Kuntzen T, Weiner B, Berical A, Charlebois P, Kuiken C (2013). Whole genome pyrosequencing of rare hepatitis C virus genotypes enhances subtype classification and identification of naturally occurring drug resistance variants. J Infect Dis.

[9] Li C, Njouom R, Pépin J, Nakano T, Bennett P, Pybus OG (2013). Characterization of full-length HCV sequences for subtypes 1e, 1h, and 1l, and a novel variant revealed Cameroon as an area in origin for genotype 1. J Gen Virol.

[10] Gu L, Tong W, Yuan M, Lu T, Li C, Lu L (2013). An increased diversity of HCV isolates were characterized among 393 patients with liver disease in China representing six genotypes, 12 subtypes, and two novel genotype 6 variants. J Clin Virol.

[11] Dunford L, Carr MJ, Dean J, Waters A, Nguyen LT, Ta Thi TH (2012). Hepatitis C virus in Vietnam: high prevalence of infection in dialysis and multi-transfused patients involving diverse and novel virus variants. PLoS One.

[12] Simmonds P (2012). SSE: a nucleotide and amino acid sequence analysis platform. BMC Res Notes.

[13] Edgar RC (2004). MUSCLE: multiple sequence alignment with high accuracy and high throughput. Nucleic Acids Res.

[14] Koletzki D, Dumont S, Vermeiren H, Peixe P, Nina J, Camacho RJ (2009). Full genome sequence of three isolates of hepatitis C virus subtype 4b from Portugal. Arch Virol.

[15] Bukh J, Purcell RH, Miller RH (1993). At least 12 genotypes of hepatitis C virus predicted by sequence analysis of the putative E1 gene of isolates collected worldwide. Proc Natl Acad Sci U S A.

[16] Li C, Lu L, Wu X, Wang C, Bennett P, Lu T (2009). Complete genomic sequences for hepatitis C virus subtypes 4b, 4c, 4d, 4g, 4k, 4l, 4m, 4n, 4o, 4p, 4q, 4r and 4t. J Gen Virol.

[17] Lu L, Nakano T, Li C, Fu Y, Miller S, Kuiken C (2006). Hepatitis C virus complete genome sequences identified from China representing subtypes 6k and 6n and a novel, as yet unassigned subtype within genotype 6. J Gen Virol.

[18] Xia X, Zhao W, Tee KK, Feng Y, Takebe Y, Li Q (2008). Complete genome sequencing and phylogenetic analysis of HCV isolates from China reveals a new subtype, designated 6u. J Med Virol.

[19] Noppornpanth S, Poovorawan Y, Lien TX, Smits SL, Osterhaus ADME, Haagmans BL (2008). Complete genome analysis of hepatitis C virus subtypes 6t and 6u. J Gen Virol.

[20] Wang H, Yuan Z, Barnes E, Yuan M, Li C, Fu Y (2013). Eight novel hepatitis C virus genomes reveal the changing taxonomic structure of genotype 6. J Gen Virol.

[21] Lu L, Li C, Yuan J, Lu T, Okamoto H, Murphy DG (2013). Full-length genome sequences of five hepatitis C virus isolates representing subtypes 3g, 3h, 3i and 3k, and a unique genotype 3 variant. J Gen Virol.

[22] Pybus OG, Barnes E, Taggart R, Lemey P, Markov PV, Rasachak B (2009). Genetic history of hepatitis C virus in East Asia. J Virol.

[23] Hübschen JM, Jutavijittum P, Thammavong T, Samountry B, Yousukh A, Toriyama K (2011). High genetic diversity including potential new subtypes of hepatitis C virus genotype 6 in Lao People's Democratic Republic. Clin Micro Inf.

[24] González-Candelas F, López-Labrador FX, Bracho MA (2011). Recombination in hepatitis C virus. Viruses.

[25] Kapoor A, Simmonds P, Cullen JM, Scheel TKH, Medina JL, Giannitti F (2013). Identification of a pegivirus (GB virus-like virus) that infects horses. J Virol.

[26] Kapoor A, Simmonds P, Scheel T, Hjelle B, Cullen J, Burbelo P (2013). Identification of rodent homologs of hepatitis C virus and pegiviruses. MBio.

[27] Quan P-L, Firth C, Conte JM, Williams SH, Zambrana-Torrelio CM, Anthony SJ (2013). Bats are a major natural reservoir for hepaciviruses and pegiviruses. Proc Natl Acad Sci U S A.

[28] Drexler JF, Corman VM, Müller MA, Lukashev AN, Gmyl A, Coutard B (2013). Evidence for novel hepaciviruses in rodents. PLoS Pathog.

[29] Choo QL, Richman KH, Han JH, Berger K, Lee C, Dong C (1991). Genetic organization and diversity of the hepatitis C virus. Proc Natl Acad Sci U S A.

[30] Inchauspe G, Zebedee S, Lee DH, Sugitani M, Nasoff M, Prince AM (1991). Genomic structure of the human prototype strain H of hepatitis C virus: comparison with American and Japanese isolates. Proc Natl Acad Sci U S A.

[31] Kato N, Hijikata M, Ootsuyama Y, Nakagawa M, Ohkoshi S, Sugimura T (1990). Molecular cloning of the human hepatitis C virus genome from Japanese patients with non-A, non-B hepatitis. Proc Natl Acad Sci U S A.

[32] Takamizawa A, Mori C, Fuke I, Manabe S, Murakami S, Fujita J (1991). Structure and organization of the hepatitis C virus genome isolated from human carriers. J Virol.

[33] Okamoto H, Kojima M, Sakamoto M, Iizuka H, Hadiwandowo S, Suwignyo S (1994). The entire nucleotide sequence and classification of a hepatitis C virus isolate of a novel genotype from an Indonesian patient with chronic liver disease. J Gen Virol.

[34] Bracho MA, Saludes V, Martró E, Bargalló A, González-Candelas F, Ausina V (2008). Complete genome of a European hepatitis C virus subtype 1g isolate: phylogenetic and genetic analyses. Virol J.

[35] Okamoto H, Okada S, Sugiyama Y, Kurai K, Iizuka H, Machida A (1991). Nucleotide sequence of the genomic RNA of hepatitis C virus isolated from a human carrier: comparison with reported isolates for conserved and divergent regions. J Gen Virol.

[36] Kato T, Furusaka A, Miyamoto M, Date T, Yasui K, Hiramoto J (2001). Sequence analysis of hepatitis C virus isolated from a fulminant hepatitis patient. J Med Virol.

[37] Okamoto H, Kurai K, Okada S, Yamamoto K, Lizuka H, Tanaka T (1992). Full-length sequence of a hepatitis C virus genome having poor homology to reported isolates: comparative study of four distinct genotypes. Virology.

[38] Murakami K, Abe M, Kageyama T, Kamoshita N, Nomoto A (2001). Down-regulation of translation driven by hepatitis C virus internal ribosomal entry site by the 3' untranslated region of RNA. Arch Virol.

[39] Nakao H, Okamoto H, Tokita H, Inoue T, Iizuka H, Pozzato G (1996). Full-length genomic sequence of a hepatitis C virus genotype 2c isolate (BEBE1) and the 2c-specific PCR primers. Arch Virol.

[40] Li C, Cao H, Lu L, Murphy D (2012). Full-length sequences of 11 hepatitis C virus genotype 2 isolates representing five subtypes and six unclassified lineages with unique geographical distributions and genetic variation patterns. J Gen Virol.

[41] Noppornpanth S, Lien TX, Poovorawan Y, Smits SL, Osterhaus ADME, Haagmans BL (2006). Identification of a naturally occurring recombinant genotype 2/6 hepatitis C virus. J Virol.

[42] Samokhvalov EI, Hijikata M, Gylka RI, Lvov DK, Mishiro S (2000). Full-genome nucleotide sequence of a hepatitis C virus variant (isolate name VAT96) representing a new subtype within the genotype 2 (arbitrarily 2k). Virus Genes.

[43] Martró E, Valero A, Jordana-Lluch E, Saludes V, Planas R, González-Candelas F (2011). Hepatitis C virus sequences from different patients confirm the existence and transmissibility of subtype 2q, a rare subtype circulating in the metropolitan area of Barcelona, Spain. J Med Virol.

[44] Sakamoto M, Akahane Y, Tsuda F, Tanaka T, Woodfield DG, Okamoto H (1994). Entire nucleotide sequence and characterization of a hepatitis C virus of genotype V/3a. J Gen Virol.

[45] Yamada N, Tanihara K, Mizokami M, Ohba K, Takada A, Tsutsumi M (1994). Full-length sequence of the genome of hepatitis C virus type 3a: comparative study with different genotypes. J Gen Virol.

[46] Chayama K, Tsubota A, Koida I, Arase Y, Saitoh S, Ikeda K (1994). Nucleotide sequence of hepatitis C virus (type 3b) isolated from a Japanese patient with chronic hepatitis C. J Gen Virol.

[47] Tokita H, Okamoto H, Iizuka H, Kishimoto J, Tsuda F, Lesmana LA (1996). Hepatitis C virus variants from Jakarta, Indonesia classifiable into novel genotypes in the second (2e and 2f), tenth (10a) and eleventh (11a) genetic groups. J Gen Virol.

[48] Chamberlain RW, Adams N, Saeed AA, Simmonds P, Elliott RM (1997). Complete nucleotide sequence of a type 4 hepatitis C virus variant, the predominant genotype in the Middle East. J Gen Virol.

[49] Timm J, Neukamm M, Kuntzen T, Kim AY, Chung RT, Brander C (2007). Characterization of full-length hepatitis C virus genotype 4 sequences. J Viral Hepat.

[50] Hmaied F, Legrand-Abravanel F, Nicot F, Garrigues N, Chapuy-Regaud S, Dubois M (2007). Full-length genome sequences of hepatitis C virus subtype 4f. J Gen Virol.

[51] Kuntzen T, Berical A, Ndjomou J, Bennett P, Schneidewind A, Lennon N (2008). A set of reference sequences for the hepatitis C genotypes 4d, 4f, and 4k covering the full open reading frame. J Med Virol.

[52] Demetriou VL, Kostrikis LG (2011). Near-full genome characterization of unclassified hepatitis C virus strains relating to genotypes 1 and 4. J Med Virol.

[53] Chamberlain RW, Adams NJ, Taylor LA, Simmonds P, Elliott RM (1997). The complete coding sequence of hepatitis C virus genotype 5a, the predominant genotype in South Africa. Biochem Biophys Res Commun.

[54] Bukh J, Apgar CL, Engle R, Govindarajan S, Hegerich PA, Tellier R (1998). Experimental infection of chimpanzees with hepatitis C virus of genotype 5a: genetic analysis of the virus and generation of a standardized challenge pool. J Infect Dis.

[55] Adams NJ, Chamberlain RW, Taylor LA, Davidson F, Lin CK, Elliott RM (1997). Complete coding sequence of hepatitis C virus genotype 6a. Biochem Biophys Res Commun.

[56] Zhou DXM, Chan PKS, Zhang T, Tully DC, Tam JS (2010). Sequence diversity of hepatitis C virus 6a within the extended interferon sensitivity-determining region correlates with interferon-alpha/ribavirin treatment outcomes. Virus Res.

[57] Tokita H, Okamoto H, Iizuka H, Kishimoto J, Tsuda F, Miyakawa Y (1998). The entire nucleotide sequences of three hepatitis C virus isolates in genetic groups 7-9 and comparison with those in the other eight genetic groups. J Gen Virol.

[58] Lu L, Li C, Fu Y, Gao F, Pybus OG, Abe K (2007). Complete genomes of hepatitis C virus (HCV) subtypes 6c, 6l, 6o, 6p and 6q: completion of a full panel of genomes for HCV genotype 6. J Gen Virol.

[59] Li C, Fu Y, Lu L, Ji W, Yu J, Hagedorn CH (2006). Complete genomic sequences for hepatitis C virus subtypes 6e and 6g isolated from Chinese patients with injection drug use and HIV-1 co-infection. J Med Virol.

[60] Lu L, Li C, Fu Y, Thaikruea L, Thongswat S, Maneekarn N (2007). Complete genomes for hepatitis C virus subtypes 6f, 6i, 6j and 6m: viral genetic diversity among Thai blood donors and infected spouses. J Gen Virol.

[61] Li C, Lu L, Zhang X, Murphy D (2009). Entire genome sequences of two new HCV subtypes, 6r and 6s, and characterization of unique HVR1 variation patterns within genotype 6. J Viral Hepat.

[62] Lu L, Murphy D, Li C, Liu S, Xia X, Pham PH (2008). Complete genomes of three subtype 6t isolates and analysis of many novel hepatitis C virus variants within genotype 6. J Gen Virol.

[63] Wang Y, Xia X, Li C, Maneekarn N, Xia W, Zhao W (2009). A new HCV genotype 6 subtype designated 6v was confirmed with three complete genome sequences. J Clin Virol.

[64] Lee YM, Lin HJ, Chen YJ, Lee CM, Wang SF, Chang KY (2010). Molecular epidemiology of HCV genotypes among injection drug users in Taiwan: full-length sequences of two new subtype 6w strains and a recombinant form_2b6w. J Med Virol.

[65] Bracho MA, Carrillo-Cruz FY, Ortega E, Moya A, González-Candelas F (2006). A new subtype of hepatitis C virus genotype 1: complete genome and phylogenetic relationships of an Equatorial Guinea isolate. J Gen Virol.

[66] Stuyver L, Wyseur A, Van Arnhem W, Lunel F, Laurent-Puig P, Pawlotsky JM (1995). Hepatitis C virus genotyping by means of 5'-UR/core line probe assays and molecular analysis of untypeable samples. Virus Res.

[67] Murphy DG, Willems B, Deschênes M, Hilzenrat N, Mousseau R, Sabbah S (2007). Use of sequence analysis of the NS5B region for routine genotyping of hepatitis C virus with reference to C/E1 and 5' untranslated region sequences. J Clin Microbiol.

[68] Stuyver L, Fretz C, Esquivel C, Boudifa A, Jaulmes D, Azar N (1996). Hepatitis C virus (HCV) genotype analysis in apparently healthy anti-HCV-positive Parisian blood donors. Transfusion.

[69] Ruggieri A, Argentini C, Kouruma F, Chionne P, D'Ugo E, Spada E (1996). Heterogeneity of hepatitis C virus genotype 2 variants in West Central Africa (Guinea Conakry). J Gen Virol.

[70] Tokita H, Shrestha SM, Okamoto H, Sakamoto M, Horikita M, Iizuka H (1994). Hepatitis C virus variants from Nepal with novel genotypes and their classification into the third major group. J Gen Virol.

[71] Stuyver L, Wyseur A, Van Arnhem W, Hernandez F, Maertens G (1996). Second-generation line probe assay for hepatitis C virus genotyping. J Clin Microbiol.

[72] Stuyver L, Van Arnhem W, Wyseur A, Hernandez F, Delaporte E, Maertens G (1994). Classification of hepatitis C viruses based on phylogenetic analysis of the envelope 1 and nonstructural 5B regions and identification of five additional subtypes. Proc Natl Acad Sci U S A.

[73] Morice Y, Roulot D, Grando V, Stirnemann J, Gault E, Jeantils V (2001). Phylogenetic analyses confirm the high prevalence of hepatitis C virus (HCV) type 4 in the Seine-Saint-Denis district (France) and indicate seven different HCV-4 subtypes linked to two different epidemiological patterns. J Gen Virol.

[74] Fretz C, Jeannel D, Stuyver L, Hervé V, Lunel F, Boudifa A (1995). HCV infection in a rural population of the Central African Republic (CAR): evidence for three additional subtypes of genotype 4. J Med Virol.

[75] Kalinina O, Norder H, Mukomolov S, Magnius LO (2002). A natural intergenotypic recombinant of hepatitis C virus identified in St. Petersburg. J Virol.

[76] Kalinina O, Norder H, Magnius LO (2004). Full-length open reading frame of a recombinant hepatitis C virus strain from St Petersburg: proposed mechanism for its formation. J Gen Virol.

[77] Kurbanov F, Tanaka Y, Chub E, Maruyama I, Azlarova A, Kamitsukasa H (2010). Molecular epidemiology and Interferon-sensitivity of the natural recombinant hepatitis C virus strain RF1 _ 2k / 1b. J Infect Dis.

[78] Kageyama S, Agdamag DM, Alesna ET, Leano PS, Heredia AML, Abellanosa-Tac-An IP (2006). A natural inter-genotypic (2b/1b) recombinant of hepatitis C virus in the Philippines. J Med Virol.

[79] Legrand-Abravanel F, Claudinon J, Nicot F, Dubois M, Chapuy-Regaud S, Sandres-Saune K (2007). New natural intergenotypic (2/5) recombinant of hepatitis C virus. J Virol.

[80] Yokoyama K, Takahashi M, Nishizawa T, Nagashima S, Jirintai S, Yotsumoto S (2011). Identification and characterization of a natural inter-genotypic (2b/1b) recombinant hepatitis C virus in Japan. Arch Virol.

[81] Bhattacharya D, Accola MA, Ansari IH, Striker R, Rehrauer WM (2011). Naturally occurring genotype 2b/1a hepatitis C virus in the United States. Virol J.

[82] Hoshino H, Hino K, Miyakawa H, Takahashi K (2012). Inter-genotypic recombinant hepatitis C virus strains in Japan noted by discrepancies between immunoassay and sequencing. J Med Virol.

[83] Tamura K, Peterson D, Peterson N, Stecher G, Nei M, Kumar S (2011). MEGA5: molecular evolutionary genetics analysis using maximum likelihood, evolutionary distance, and maximum parsimony methods. Mol Biol.

